# Sequencing the ocular surface microbiome: a review of methodological practices and considerations

**DOI:** 10.3389/fopht.2025.1660816

**Published:** 2025-11-20

**Authors:** Shiva Mehravaran, Mihai Pop

**Affiliations:** 1 Center for Bioinformatics and Computational Biology (CBCB), University of Maryland, College Park, MD, United States; 2 Department of Biology, School of Computer Mathematical and Natural Sciences, Morgan State University, Baltimore, MD, United States; 3 Department of Computer Science, University of Maryland, College Park, MD, United States

**Keywords:** ocular surface microbiome, microbiome sequencing, methodological standardization, minimum reporting guidelines, microbiome reproducibility, ophthalmic microbiome

## Abstract

**Purpose:**

The human ocular surface microbiome (OSM) plays a vital role in ocular health, infection prevention, and immune modulation. However, use of sequencing technology for researching the OSM is challenged by low sample biomass, high sample variability, and methodological inconsistencies. This review systematically evaluates existing literature on OSM research, identifying methodological challenges and proposing standardization strategies to enhance data quality, comparability, and clinical relevance.

**Methods:**

A comprehensive analysis of peer-reviewed studies was conducted to assess methodologies used in sequencing-based OSM research, with focus on considerations in scope: sample size, selection, choice of eye, time frame, recruitment and enrollment criteria; sample collection and handling: sampling environment, topical anesthesia, sample collection tools and ocular region; sample preservation: temperature and use of buffers; and sample analysis: DNA extraction, quantification, and sequencing approach. Advantages and limitations of different approaches were identified, and best practices for standardization were explored.

**Results:**

This review identified substantial variations in sample collection and processing methodologies, many of which are known to impact OSM composition. However, the influence of certain approaches remains unclear. Additionally, large reporting gaps were observed, as many studies failed to describe critical methodological elements, including specific sample handling procedures and sequencing parameters.

**Conclusions:**

While sequencing technologies offer valuable insights, our findings highlight the need for further investigation of different methodological approaches to determine best practices and establish standardized methodological protocols, as well as the need for standardized reporting protocols in OSM research. These standards are essential for enhancing data reliability and translating findings into clinical applications.

## 1 Introduction

The human ocular surface microbiome refers to the diverse and dynamic community of microorganisms that inhabit the cornea, conjunctiva, and tear film. The ocular surface microbiome is believed to play an important role in maintaining ocular health and preventing infections, as well as modulating immune responses and inflammation (1–3). However, various factors, such as age, sex, lifestyle, environment, and disease states may alter its composition and function (1, 4). Therefore, understanding the ocular surface microbiome and its interactions with the host and the environment is essential for developing novel strategies for diagnosis, prevention, and treatment of ocular diseases.

A key challenge in studying the ocular surface microbiome is the low biomass and high variability of the samples, which demand sensitive and reliable methods for the detection, identification, and characterization of the microbial communities (3, 5). In recent years, sequencing technologies, such as shotgun and 16S rRNA gene sequencing, have emerged as powerful tools for conducting microbiome research, as they can provide comprehensive information on the taxonomic and functional diversity of the microbiome. However, these technologies also introduce methodological challenges, such as sample collection, processing, storage, extraction, amplification, quality control, analysis, and interpretation, which may affect the accuracy and reproducibility of the results (6, 7).

In a recent review, Clougher et al. (8) synthesized current knowledge of the ocular surface microbiome in healthy and diseased states; however, they found inconsistencies among currently used methodological and analytical protocols that can impact results and hinder valid comparisons, and they highlighted the critical need for standardization. Building upon their work, the aim of this review is to provide a more in-depth systematic and critical overview of methodological choices employed in human ocular surface microbiome research and examine their implications for the findings and conclusions.

This review addresses practical aspects of conducting ocular surface microbiome research in human subjects, from broader concepts like study scope and sample size to more specific technical details like DNA extraction and sequencing approach, while discussing the advantages and limitations of different approaches. By focusing on these specific aspects, this review aims not only to identify areas where further standardization is needed, but also to serve as a methodological guide for researchers entering the field. Ultimately, it contributes to enhancing the reproducibility, comparability, and overall quality of findings in ocular surface microbiome research.

## 2 Methodology of the review

A comprehensive literature search was conducted in December 2024 using PubMed and Google Scholar, supplemented by exploring the bibliographies of relevant articles. The search was limited to English-language original research articles in humans, focusing on bacterial identification of the ocular surface microbiome through sequencing techniques.

This search yielded 89 original research articles (9–97) and 26 BioProjects in the National Library of Medicine repositories, which represents a significantly smaller body of research compared to other well-studied human microbiomes, such as the oral, vaginal, gut, and skin microbiomes which have over 200 BioProjects each. This relative paucity of research underscores the need for increased investigation into the ocular surface microbiome. Nonetheless, this limited dataset provides a unique opportunity to characterize the current landscape of research methodologies, including the prevalence of various methodological approaches and reporting practices.

**Figure 1:**
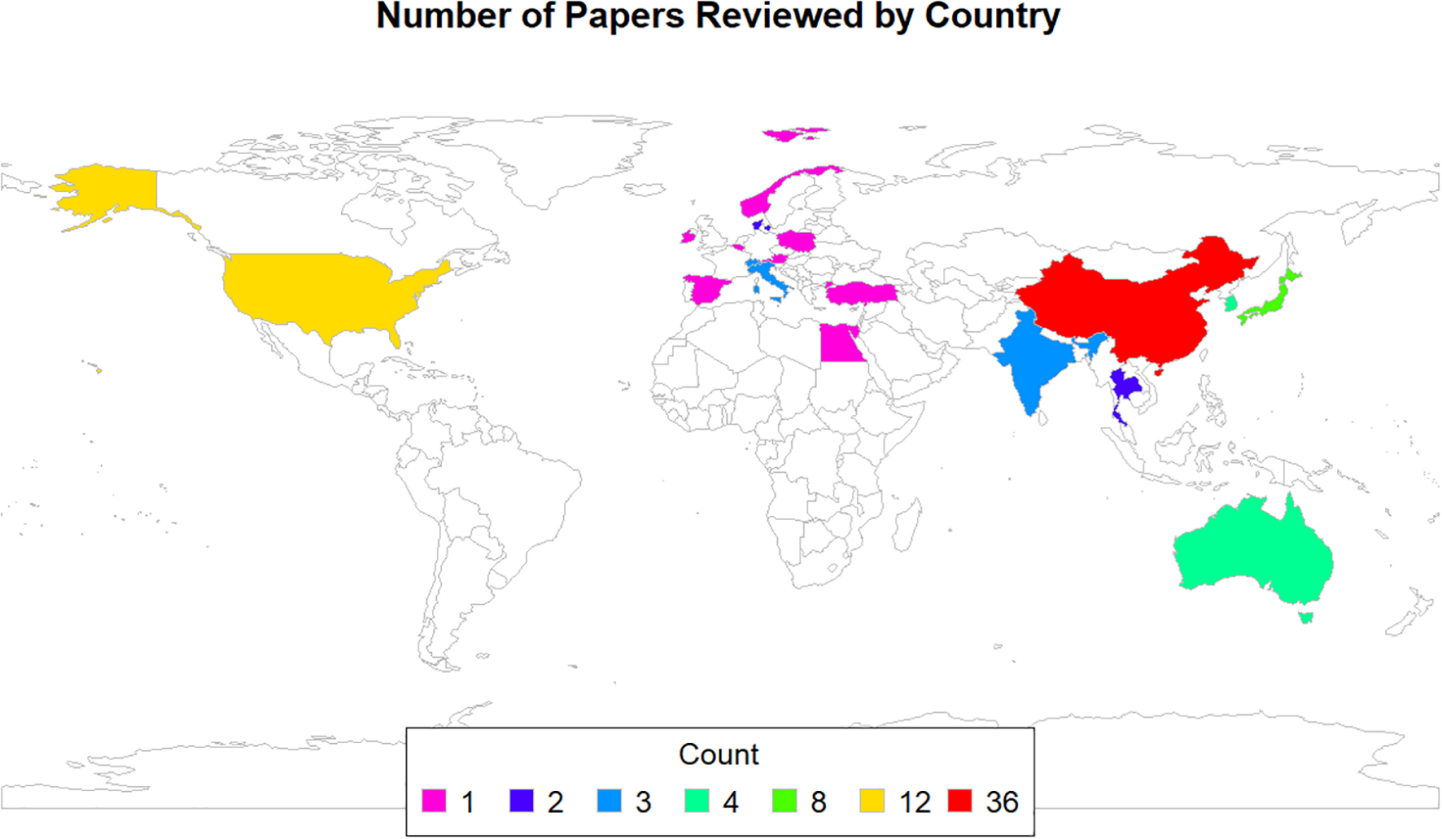
Number of papers reviewed by country (total n = 89).

In terms of country of origin, as illustrated in **Figure 1**, 36 of the articles were published by researchers in China, 12 were conducted in the USA, 8 in Japan, 4 each in Australia and Korea, 3 each in India, Italy, and Switzerland, 2 each in Denmark and Thailand, and 1 each in Austria, Belgium, Egypt, Hong Kong, Ireland, Norway, Poland, Singapore, Spain, the Gambia, and Turkey. Additionally, one study was a multicenter collaboration between Spain and Italy.

## 3 Review of methodological approaches

### 3.1 Study scope

Of the 89 studies reviewed, 66 (74.2%) employed a case-control design, and three were before-after longitudinal studies. Additionally, some studies focused exclusively on specific participant groups, with 18 studies involving only normal participants and 2 studies enrolling only disease cases.

#### 3.1.1 Sample size

The sample size can influence precision: a sample size that is too small can underpower the study and negatively affect the validity and reproducibility of the findings, while an oversized study uses more resources than necessary, and exposes more subjects to a potentially harmful experience (98).

In microbiome studies, additional considerations might be applicable to how we estimate the sample size because microbiome datasets have intrinsic features that do not apply to traditional studies. A key challenge lies in defining biologically meaningful effect sizes for the microbial community changes or individual taxa differences we aim to detect, which complicates power analysis and makes it a main barrier to robust study design in microbiome research (99–101). For ocular surface microbiome research, dealing with the low abundance microbiome necessitates exceeding the calculated estimates to account for individual differences, technical variability, and subgroup analyses. Furthermore, the low biomass of the ocular surface can lead to a higher proportion of samples with insufficient DNA yield for downstream analyses. This “sample loss” needs to be accounted for when determining the initial sample size for a study.

Sample size calculations, or the lack thereof, were explicitly reported in only 17 (19.1%) of the articles reviewed. Among the 89 papers, 13 (14.6%) described their sample size calculation approach. For instance, four studies utilized Micropower, an R package developed by Kelly et al. (100), to estimate power and pairwise sample sizes based on permutational multivariate analysis of variance (PERMANOVA). These studies determined that low-abundance 16S rRNA datasets required a minimum sample size of 30 to achieve a discriminant power of 0.8 with a significance level of 0.05 for detecting statistically significant differences in alpha or beta diversity of the microbiome composition between groups (39, 64, 80, 92). Other tools used for sample size calculation included StateMate Software version 2.0 (GraphPad Inc., San Diego, CA) (15), Easy R (EZR, version 1.41) (35), and G*Power software (version 3.1.9.7) (70) which calculated a required sample size of 42 patients and 64 controls, based on an effect size of 0.5 (with no reference to a specific metric), alpha error of 0.05, and power of 0.80. Additionally, two studies employed species accumulation curves from prior ocular microbiome studies, determining that 20–45 samples were sufficient to detect >80-85% of microbial species (85, 95). Population proportion methods were also used, with one study deriving a sample size of 21 based on a 6% margin of error and 90% confidence interval to compare abundance of pathogenic bacteria among three groups, while another study, which compared the microbial composition among four groups, calculated a sample size of 20 eyes per group using a formula comparing two independent means and standard deviations from a previous study (55, 75).

Reasons for omitting sample size calculations included the pilot nature of the study, which aimed to provide preliminary data for future research, or the descriptive nature of the study, which lacked a formal null hypothesis (41, 43, 71, 83). Notably, 73 articles (82.0%) did not perform sample size calculations or provide a justification for their chosen sample size. Nonetheless, many of these studies acknowledged their small sample size as a limitation, highlighting this as a recurring challenge in the field (12, 28, 35–37, 43, 46, 47, 50, 57, 61, 65, 78, 84, 89, 90, 93).

**Figure 2:**
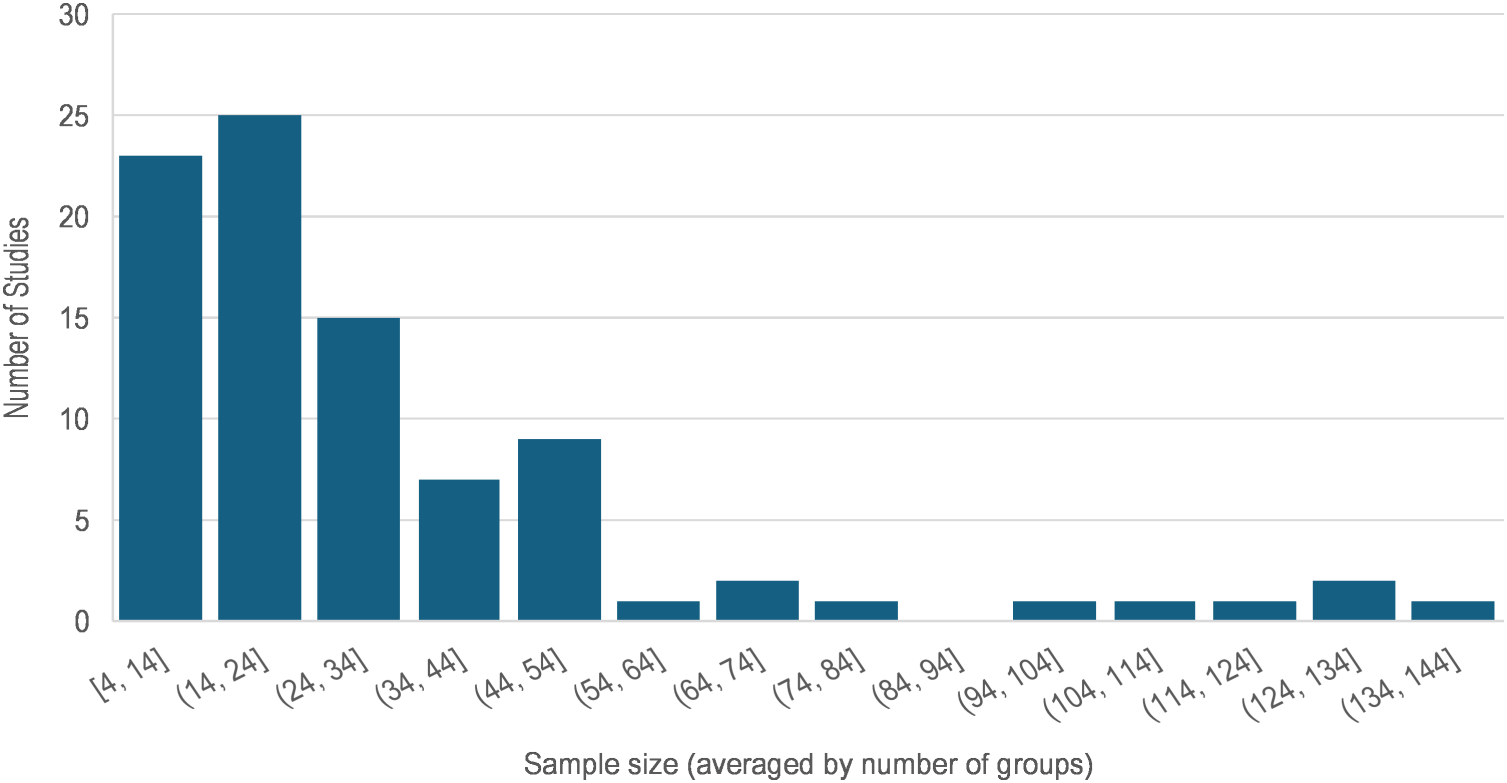
Frequency distribution of the sample sizes in the 89 reviewed studies.

The total number of participants enrolled in each study ranged between 4 and 260 human subjects (median = 48), and the sample size for groups (e.g. in case-control studies) ranged between 4 and 137 (median = 22). **Figure 2** illustrates the sample size variability across these 89 articles where the average group size was used for multigroup studies. Of the 71 (79.8%) studies that enrolled two or more groups of participants, 67 enrolled healthy normal individuals as the control group. The case and control groups were the same size in 15 of these studies. In 26 studies, the sample size of the control group was 2% to 339% larger than that of the cases. In the remaining 26 studies, the sample size of the normal control group was 3% to 76% smaller than that of the cases. Nonetheless, comparison of the sample sizes between case and control groups showed no statistically significant difference (paired t-test p = 0.268).

#### 3.1.2 Choice of eye

While the choice of left or right eye for sampling might seem inconsequential, the decision to sample unilaterally or bilaterally has significant implications for study design and analysis. This distinction is crucial because most statistical methods assume that samples are independent. If these assumptions are violated, such as when analyzing data from both eyes of the same individual, the calculated statistics can be misleading, potentially leading to inflated Type I error rates (false positives) or decreased statistical power (102, 103).

**Figure 3:**
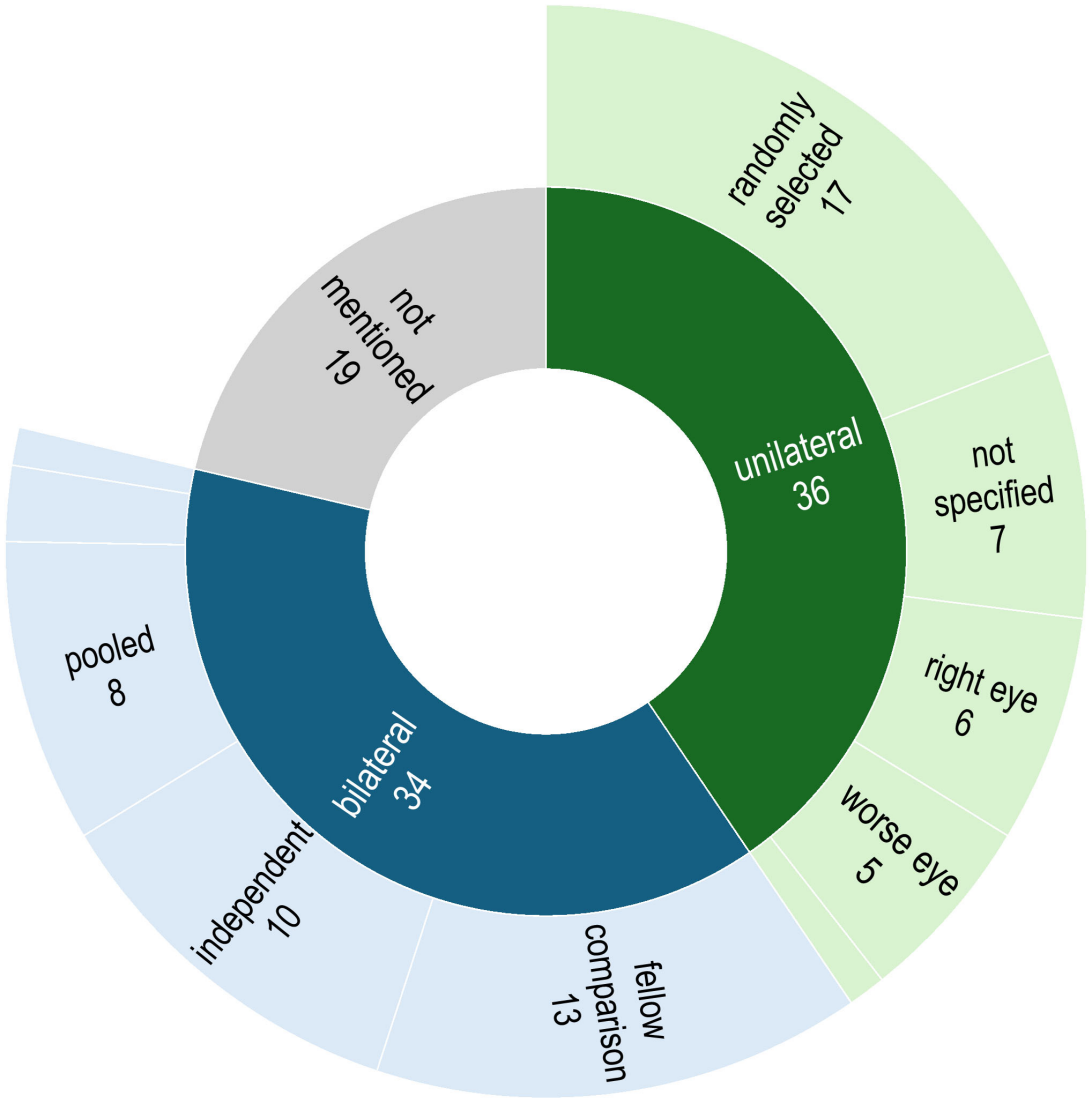
Distribution of unilateral or bilateral sampling approach.

In this review, the choice of unilateral or bilateral sampling approach was either explicitly stated or could be deduced in 70 (78.7%) of the articles (**Figure 3**). Of these, 36 studies performed sampling from only one eye; this was either a randomly-chosen eye (n = 17), the right eye (n =6), the left eye (n = 1), the eye receiving treatment (n = 5), or the selection was not specified (n = 7). In 34 studies, samples were obtained from both eyes of each participating individual; these were used for performing fellow eye comparisons (n = 12), counted as independent samples to increase the overall sample size (n = 11), pooled as one sample (n =8), collected separately to perform downstream analysis with the one generating a higher DNA yield (n = 1), or it was unclear whether they were treated independently or pooled (n = 2). In the remaining 19 articles (16.9%), it was not clear whether sampling was done unilaterally or bilaterally.

#### 3.1.3 Time frame

For the gut (104, 105), skin (106), and respiratory system (107) microbiome, studies have observed significant seasonal variations influenced by factors such as diet, lifestyle and behavior changes (e.g. stress, sleep patterns, and indoor/outdoor physical activity), and environmental conditions. Similarly, the ocular surface microbiome is a dynamic ecosystem which can be influenced by internal and external factors over time. Diurnal variations, such as eyelid closure during sleep, tear film dynamics, and exposure to environmental factors (e.g., pollutants, allergens) may significantly impact the microbiome throughout the day. Seasonal changes in environmental factors (e.g., humidity, temperature, pollen levels) can also affect the ocular microbiome. Furthermore, host circadian rhythms can influence tear production, immune function, and potentially the composition of the ocular microbiome. Thus, sampling time is an important factor in ocular microbiome research, and its clear reporting is essential for analyzing how the microbiome composition and function change over different time scales, controlling for the potential influence of time variations, and making valid comparisons between studies.

**Figure 4:**
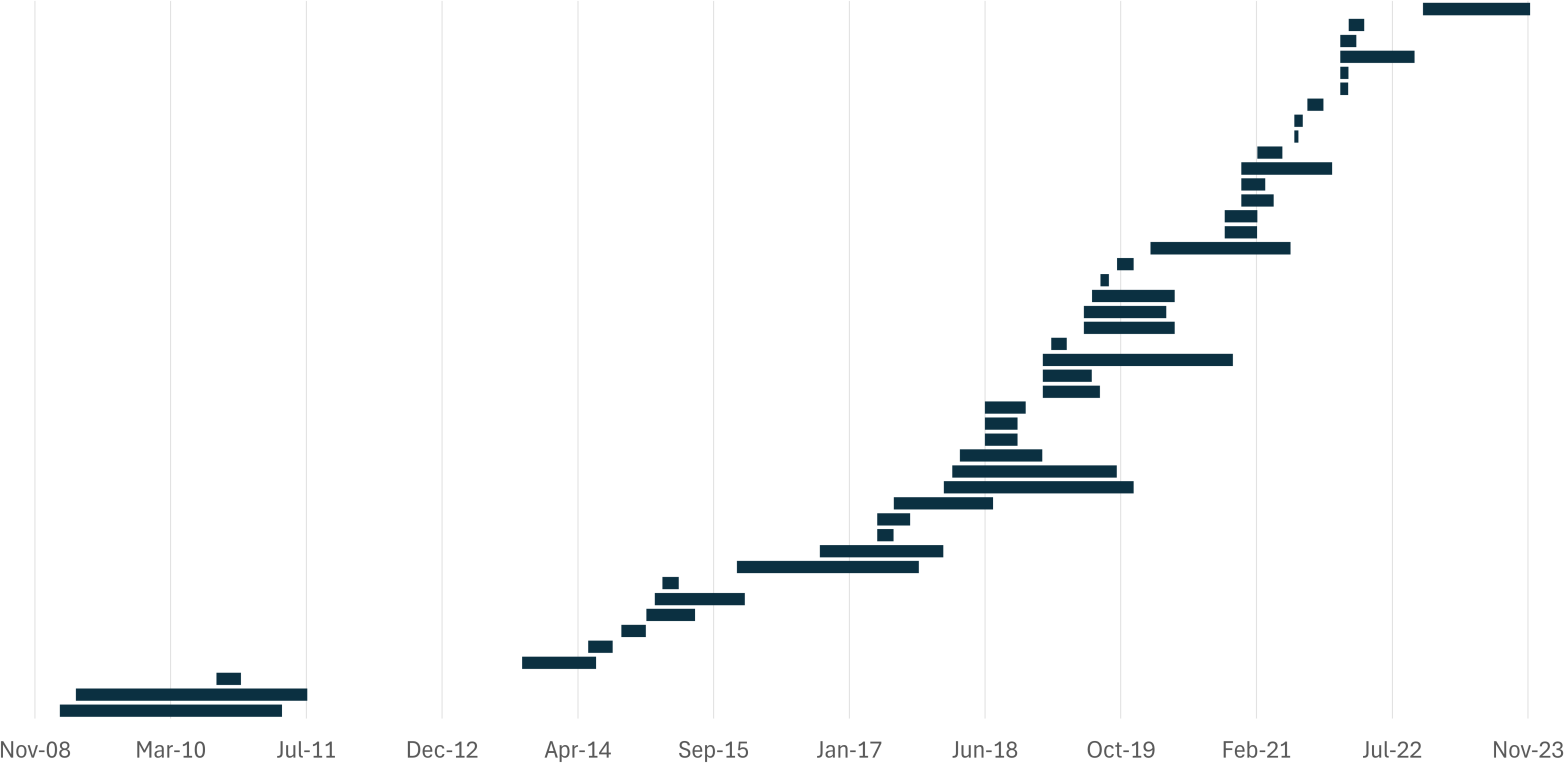
Timeline of the 45 (out of 89) studies reporting their start and end dates.

The study time frame was specified as the start and end month in 45 articles (50.6%), 3 articles reported the duration, and 3 reported the years in which the study was done. In the 48 studies that provided information about the study duration, the sampling periods ranged from 2 weeks to 28 months (median = 4.5 months) (**Figure 4**). Although very few of the studies were longitudinal, 10 studies had sampling periods between 3 and 6 months, 11 were conducted over 6 to 12 months, and 10 had a time frame longer than 12 months. The remaining 41 studies (46.1%) did not provide information about the study duration, suggesting a cross-sectional study design at an undefined point in time. Moreover, the sampling time of the day was specified in only four articles (4.5%), all of which stated that samples were collected in the morning.

#### 3.1.4 Recruitment and enrollment criteria

Recruitment and enrollment criteria are foundational to study design and analysis. They define the study population, and they are essential to control bias, enhance validity, and ensure generalizability. Common inclusion criteria, like age ranges, separate distinct groups (e.g., adults vs. children) with potentially significant differences in the study variable. When recruiting patients with a disease condition of interest, such as diabetes or dry eye, the inclusion criteria are often based on established diagnostic definitions to focus the research on a well-defined population. For example, the Ocular Surface Disease Index is used to include patients with dry eye disease (31), or to rule out the condition from the normal controls (19, 43). Exclusion criteria, on the other hand, serve to remove individuals with factors that could distort the results. This might involve excluding participants with pre-existing conditions that could interact with the studied intervention or those taking medications that might bias the findings.

Among the reviewed studies, 63 (70.8%) applied convenience sampling and recruited their cases from clinics and hospitals. Specifically, participants were recruited from those visiting clinics and hospitals for an eye exam (n = 52), those undergoing surgery (n = 9), hospitalized patients (n = 1), and medical staff (n = 1). Additionally, some studies recruited participants from the general population, either through community cohorts (n = 4) or community screening programs (n = 1), as well as through internet surveys and advertisements (n = 2). A few studies used a combination of recruitment methods, such as recruiting from both inpatients and outpatient, medical school students, and retirement communities (n = 3). The recruitment pool was not mentioned or unclear in the remaining 16 articles.

Exclusion criteria were provided in 82 (92.1%) of the reviewed articles. The most common exclusion criteria were use of systemic and/or topical antibiotics (n = 54), contact lens wear (n = 48), ocular (or ocular surface) disease and/or infection (n = 47), systemic disease (n = 40), use of ocular medications (n = 37), history of ocular surgery (n = 43) or trauma (n = 23), pregnancy (n = 17) and/or lactating (n = 11), use of corticosteroids or immunosuppressants (n = 20). Some articles also included skin disease and/or infection (n = 4), tobacco use (n = 9), ocular allergies (n = 3), environmental considerations such as being a healthcare worker or having spent a significant time in a hospital (n = 2), mental illness (n = 1), intravenous drug injection (n = 1), and certain dietary habits (vegetarian or vegan) (n = 1).

### 3.2 Sample collection and handling

#### 3.2.1 Sampling environment

In microbiome research, adhering to aseptic techniques is crucial to minimize contamination, especially when dealing with low microbial biomass, such as the ocular surface microbiome (108). Sampling should ideally be conducted in a clean or sterile environment, to reduce the introduction of environmental contaminants. The inclusion of negative controls - such as unused sampling tools exposed to the same collection environment - help identify and account for potential contamination introduced during sampling, or during manufacturing, packaging, or handling.

The sample collection environment could be determined in only 25 (28.1%) of the articles. The environments described included ophthalmic treatment rooms sterilized with ultraviolet light (n = 8), clean rooms (n = 7), sterile operating rooms (n = 2), a clean prior fumigated room (n = 1), and rooms with appropriate lighting, temperature, and humidity (n = 2). Additionally, two studies performed sample collection in the operating room during surgery, one in a lab or ophthalmology office, one in the field, and one where participants collected their own samples at home. In the remaining 64 (71.2%) articles, the sample collection environment was not mentioned.

The inclusion of unused collection tools (e.g. swabs) as negative environmental controls was mentioned in only 42 studies (47.2%), including 17 of the 25 studies that had a description of their sample collection environment. However, reports of the results with these negative controls were found in only 10 articles. Some carried out the analyses only up to the DNA extraction stage (n = 3). An example is the study by Ham et al. (24) who reported that their unused dry cotton swabs yielded no significant amount of DNA (below 0.05 ng DNA μL-1) and thus, the samples were not amplified. Similarly, Liang et al. (50) found no bacterial DNA in the negative control swabs, although it is not clear how DNA was quantified. In some cases, the researchers ruled out contamination by checking PCR amplification results in their negative controls (n = 5). One example is the study by Graham et al. (10) who confirmed that the conventional broad-range 16S rDNA PCR products of their negative controls produced no visible bands on gel electrophoresis. In the remaining two studies, full analysis was conducted on the negative controls, and the operational taxonomic units (OTUs) or amplicon sequence variants (ASVs) found in these controls were removed from the samples for contaminant filtering. On the other end of the spectrum, Zhou et al. (14) did their sample collection in the field, and although their methods section does not mention collecting negative controls during sampling, their results indicate running some through the complete set of analyses and finding *Ralstonia* to be the major taxon in their negative controls, and thus, they were not able to confirm whether its presence in 80% of the participant samples was due to contamination or part of the ocular flora.

#### 3.2.2 Topical anesthesia

Sample collection from the ocular surface can be a challenge because the eye is a very sensitive organ and shows strong reflexes such as blinking and tear secretion in response to external stimuli such as dust and light. With a nerve density of 300–400 times greater than that of the skin, the cornea is one of the most sensitive tissues in the human body (109). As such, instilling proparacaine hydrochloride 0.5% or tetracaine hydrochloride 0.5% ophthalmic drops is a common technique for achieving topical anesthesia before performing ophthalmic procedures, even non-surgical ones (110–112). However, the antibacterial effects of proparacaine have long been known using culture techniques (113, 114). Using culture-independent techniques, Shin et al. (18) observed that using proparacaine before sampling leads to lower diversity levels, while Delbeke et al. (65) who conducted a contralateral control study, found that it specifically effects *Cutibacterium*.

**Figure 5:**
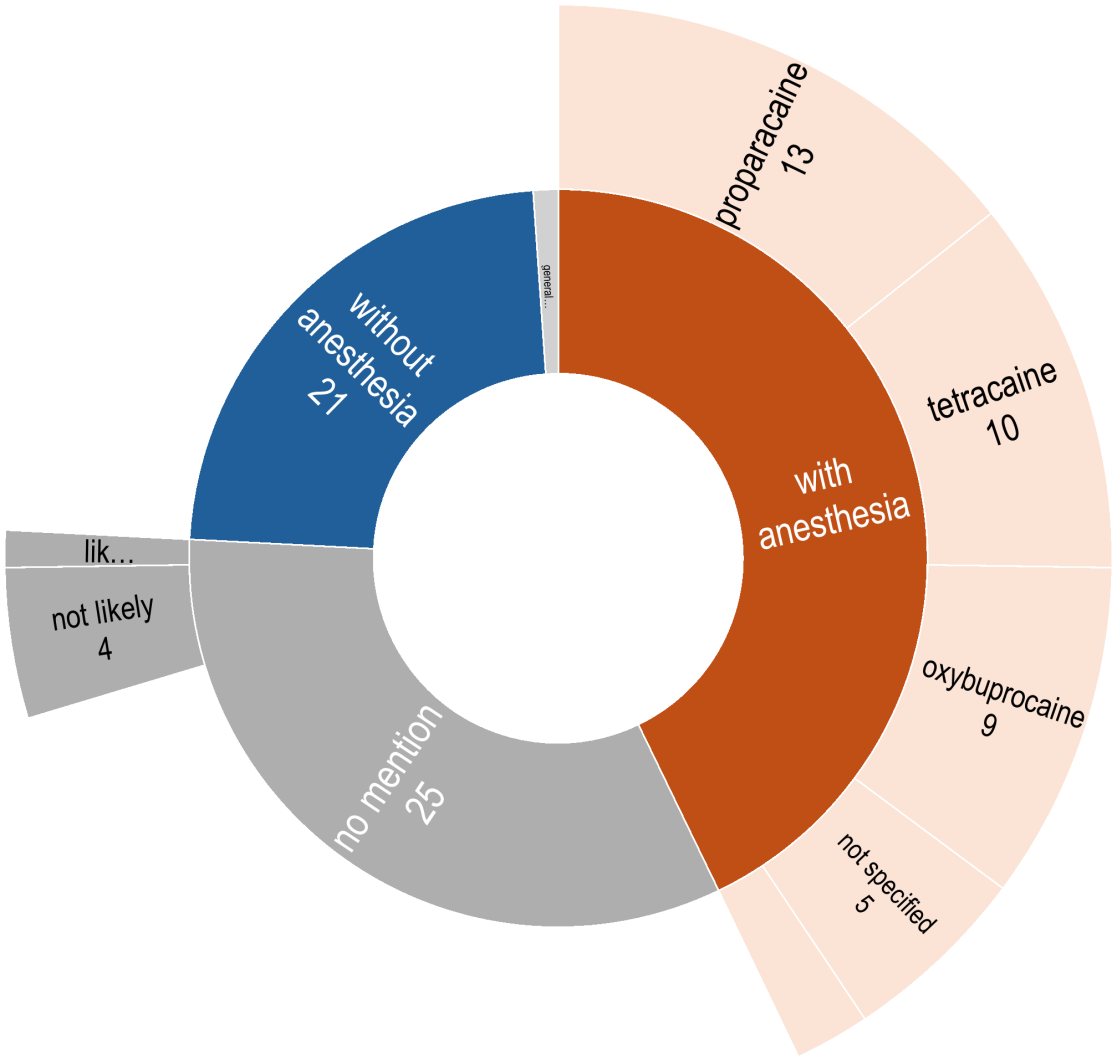
Distribution of descriptions regarding the administration of anesthesia prior to ocular surface sampling.

As illustrated in **Figure 5**, information regarding the use of topical anesthetics prior to sampling was available in 60 (67.4%) studies; in 39 studies anesthetics were used, while in 21 studies, the authors explicitly stated that no anesthetics were administered. Proparacaine was the most frequently administered topical anesthetic (n=13), followed by tetracaine (n=10), oxybuprocaine (n=9), and Alcaine (n = 2). In the remaining five studies, sample collection was done after instilling anesthetics, but the actual preparation was not stated. In the study by Cavuoto et al. (22), sampling for the children was performed under general anesthesia, although the use of anesthetic agent is not mentioned for their adult participants. For the remaining 28 studies (31.4%), it was not clear whether anesthetics were administered before ocular surface sampling.

#### 3.2.3 Collection tools and ocular region

Research on the effect of collection tools and sampling approaches in ocular surface microbiome is sparse. Herzog et al. (115) compared standard cotton versus flocked nylon swabs and observed that the choice can influence results. Büttner et al. (116) reported that pre-moistened swabs increase the rate of Gram-positive bacterial detection in their study of feline conjunctival microbiota. Ozkan et al. (117) suggest there can be significant differences in bacterial richness, diversity, and composition between tissue samples and swab samples. Comparing 12 different ocular regions using culture-based methods, Fahmy et al. (118) found that *Staphylococcus albus* and corynebacteria were less likely to be recovered from the bulbar conjunctiva. In terms of the pressure applied while swabbing, Dong et al. (11) suggested that deep (more pressure) sampling leads to a higher abundance of Proteobacteria, while soft sampling was associated with Firmicutes and Actinobacteria.

In the reviewed articles, a variety of tools were employed for sample collection. The most common tool was sterile swabs (n = 70) which varied by material (cotton, nylon, or flocked), tip fiber arrangement (spun or flocked), and preparation (dry, pre-moistened, or soaked) (**Table 1**). Less commonly used collection tools included paper (n = 5), membrane (n = 5), pipette with 1.0 ml of saline rinse (n = 1), and capillary tube (n = 2). One of the latter cases is the study by Pal et al. (72) who suggested that tear samples collected with capillary tubes can produce results comparable to conjunctival swabs. Three studies compared various tools, including swabs. For example, Katzka et al. (47) compared conjunctival swabbing using three different materials: calcium alginate, cotton, and Weck-Cel cellulose. They found that calcium alginate swabs yielded results most similar to corneal epithelial biopsies, while Weck-Cel cellulose sponges differed the most. Chen et al. (80) reported differences in alpha diversity and microbial composition between pre-moistened cotton swabs and tear test paper strips. In the remaining articles, tissue was collected during surgery (n = 1), saline eye wash was collected in a tube (n = 1), or the tissue extraction tool/method was not described (n = 1).

**Table 1:** Specifics of the swabs as described in 70 studies.

Material	Tip arrangement	Preparation	N
calcium alginate		dry	1
calcium alginate			1
cellulose sponge			1
cotton			32
cotton		dry	6
cotton		saline	1
cotton	flocked		1
nylon	flocked		7
nylon	flocked	PBS	1
nylon	flocked	wet	1
	flocked	moist	1
nylon			3
		oxybuprocaine	1
		saline	1
	flocked		7
no swab specifics			11

Of the 73 studies that used swabs, 58 provided descriptions of their various sampling techniques. The swabbed conjunctival regions included the general conjunctival sac surfaces (n = 19), or explicitly the palpebral conjunctiva (n = 2), palpebral conjunctiva and fornix (n = 9), the bulbar conjunctiva and fornix (n = 5), just the fornix (n = 15), or just the bulbar conjunctiva (n = 4); both the superior and inferior conjunctival surfaces (n = 22) or just the inferior conjunctiva (n = 40), and the choice to include the caruncle (n = 8) or the lid margin (n = 2). Descriptions also included the number of times the swab was swept across the conjunctival surface (n = 19), the level of pressure exerted during swabbing (n = 7), the direction of swabbing (n = 12), and precautions to avoid contamination or contact with other regions (n = 4).

### 3.3 Sample preservation

Sample preservation is a critical step in microbiome studies to maintain the original microbial composition, which can change due to microbial growth, death, or metabolic activity, potentially lead to inaccurate results. Proper preservation methods are essential, especially for studies requiring the analysis of samples collected at different times and locations. This ensures long-term storage without compromising their quality.

Several studies in other fields of microbiome research have aimed to determine the effect of sample preservation factors such as stabilization buffers, storage time, and temperature on microbial communities; however, their methods widely vary, and their results are inconclusive. For example, Lyons et al. (119) suggested that freezing samples at -80°C is the best alternative when immediate DNA extraction is not feasible. Menke et al. (120) found that immediate freezing without buffer is preferable for fecal swabs, while Hang et al. (121) observed greater changes in microbial mock communities at higher temperatures. Kool et al. (122) reported that stabilization buffers can limit microbial overgrowth at room temperature but may affect the relative abundance of certain bacteria compared to immediate freezing.

Information about the use of sample transport media was provided in 71 (79.8%) of the reviewed articles. In 38 of these articles, samples were placed in tubes with no transport media or preservation solution. In the other 32 studies, the collected samples were placed in various types of media such as lysis solution (n = 6), phosphate-buffered saline (n = 5), liquid Amies (n = 4), DNA protective solution (n = 4), DNA/RNA Shield (n = 3), DNase-Free double-distilled water (n = 1), Stuart’s transport medium (n = 1), RNAlater (n = 1), thioglycollate broth (n =1), normal saline (n = 1), swab solution (100 mM Tris-HCl [pH 8.0], 30 mM EDTA [pH 8.0], and 0.5% sodium dodecyl sulfate) (n = 1), ST solution (0.15 M NaCl and 0.1% Tween‐20) (n = 1), stringent wash 1 solution and glass beads (n = 1), and unspecified “transport medium” (n = 2). The quantity of the transport medium, as reported in 15 of these articles, ranged between 250 µl (Dacron swab placed in RNAlater) and 1 ml (cotton swab placed in phosphate-buffered saline).

Temperature considerations for the samples were expressed in 65 (73.0%) articles for at least one of the following time frames: immediate post-collection (n = 26), short-term and/or long-term storage (n = 59), and during transport (n = 5). Immediate post-collection temperatures were +4° (n = 3), -18° (n = 1), -20° (n = 4), and “on ice” (n = 5), and the durations ranged between 1 to 6 hours. Short-term and/or long-term storage temperatures were -18 (n = 1), -20 (n = 10), -70° (n = 1), and -80 (n = 39). Few articles specified more than two time frames and/or their maximum durations. Examples include the study by Ozkan et al. (19), in which samples were immediately frozen at -20°C using a LabTop Cooler (Thermo Fisher Scientific, Coralville, MA, USA), transferred to a -80°C freezer within an hour, and processed within 6 months. In another study by Ozkan et al. (26), collected tissue samples were immediately frozen at -20°C and transported to the lab on dry ice to be stored at -80°C within 48 hours. Delbeke et al. (65) initially froze the samples at -18°C and transferred them to a -80°C freezer within two weeks. Mohamed et al. (53) stored samples at +4°C for the first day and -20°C until transfer, while Doan et al. (16) placed samples on ice immediately, transferred them to the lab within 6 hours, and stored there at -80°C.

### 3.4 Sample analysis

#### 3.4.1 DNA extraction

In human fecal microbiome, the choice of the DNA extraction methods has been found to have a large impact on the results of metagenomic analyses, due to variations in cell lysis efficiency and the introduction of biases (123–126). These factors, along with DNA contamination and the presence of inhibitors, are important considerations in any microbiome study (127).

However, ocular surface microbiome research presents unique challenges. The inherently low bacterial biomass of ocular samples often results in low DNA yields, exacerbating the problems caused by inefficient lysis. Furthermore, ocular samples may contain a high proportion of host DNA, and the prevalence of Gram-positive bacteria with their robust cell walls necessitates effective lysis strategies. Another consideration is components of the tear film or other ocular secretions that may act as PCR inhibitors. Given these challenges, DNA extraction methods for ocular samples require optimization to maximize bacterial DNA yield, minimize host DNA contamination, and effectively lyse both Gram-negative and Gram-positive bacteria while removing potential inhibitors, ensuring accurate microbiome characterization.

Various DNA extraction methods were utilized in the reviewed papers. In 78 studies (87.6%), a commercial kit (**Table 2**); of these, 52 stated the extraction was done in accordance with the manufacturer’s instructions, and 11 applied modifications such as adding a lysis step (n = 7), RNA digestion step (n = 2), or optimization for high throughput (n = 1). Five studies described their custom/homemade protocols, and in the remaining 6 articles, the DNA extraction method was not mentioned or not clear.

**Table 2:** List of various DNA/RNA extraction kits and their manufacturers used in 78 of the reviewed studies.

Manufacturer	Kit name	N of articles	Ref. #
Bio 101	FastDNA Spin Kits for Soil	1	(11)
Epicenter	MasterPure Complete DNA and RNA purification Kit	5	(21, 35, 50, 60, 72)
IngeniGen XMK Biotechnologies	DNA extraction kit	1	(68)
iNtRON	i-genomic Soil DNA Extraction Mini Kit	2	(25, 71)
Kurabo	Quick Gene DNA tissue kit S	1	(57)
Macherey-Nagel	NucleoSpin 96 Soil Kit	2	(58, 62)
	NucleoSpin Tissue XS kit	7	(20, 27, 43, 44, 83, 86, 96,)
Magen	HiPure soil DNA kit	1	(79)
MinkaGene	MinkaGene Bacterial DNA Kit	2	(32, 33)
MoBio	DNeasy PowerSoil Kit	1	(89)
	PowerMag Microbiome kit	1	(24)
	PowerMax Soil DNA Isolation Kit	3	(30, 41, 49)
	PowerSoil DNA Isolation Kit	8	(12, 15, 19, 23, 28, 29, 45, 52)
MP Biomedicals	DNA Extraction Kit	1	(59)
Omega	E.Z.N.A. MicroELute Genomic DNA kit	1	(88)
	MicroElute Genomic DNA Kit	4	(18, 31, 75, 92)
Omega Bio-Tek	DNA extraction kit	1	(55)
	E.Z.N.A. MicroELute Genomic DNA kit	2	(61, 94)
	OMEGA Soil DNA Kit	3	(81, 82, 93)
Qiagen	Allprep PowerViral DNA/RNA Kit	1	(64)
	DNeasy Blood & Tissue Kit	1	(17)
	DNeasy PowerLyzer PowerSoil	1	(78)
	PowerSoil	2	(70, 74)
	QIAamp DNA Microbiome Kit	5	(63, 76, 87, 97, 98)
	QIAamp DNA Mini Kit	2	(56, 73)
	QIAamp MinElute Virus Spin Kit	1	(69)
	QIAamp UCP Pathogen Mini Kit	2	(37, 47)
	QIAamp Viral RNA Mini Kit	1	(10)
	RNeasy Plus Micro RNA Isolation Kit	1	(16)
	RNeasy PowerMicrobiome	1	(66)
	QiaAmp Microbial DNA Isolation Kit	1	(84)
	DNeasy PowerSoil	4	(39, 65, 67, 80)
ThermoFisher Scientific	PureLink Microbiome DNA Purification Kit	1	(34)
Tiangen	DNA Extraction Kit	1	(26)
Zymo Research	DNA Miniprep	1	(48)
	Quick-DNA Fecal/Soil Microbe Miniprep	1	(54)
	ZR-Duet DNA/RNA MiniPrep	1	(77)
	ZymoBIOMICS DNA Miniprep	1	(90)
	ZymoBIOMICS 96 MagBead DNA	1	(95)
Unspecified	Unspecified	1	(51)
Total		78	

#### 3.4.2 Checking the extracted DNA

In microbiome research, the quantity and quality of DNA is typically checked at certain key points: 1) after DNA extraction to ensure that the DNA is suitable for downstream applications like PCR and sequencing; 2) during library construction to ensure that the correct amount of DNA is used for creating the libraries; and 3) after library preparation to verify the concentration of the final libraries and prevent issues related to overloading or underloading the sequencer, which can affect the overall data quality. While a successful study may not require very high yields, the extracted DNA must be of sufficient quality and quantity for downstream analysis to be informative.

The consensus in ocular surface microbiome research is that samples are low biomass, and transparent reporting of DNA yield and quality metrics is crucial for evaluating the success of DNA extraction and interpreting microbiome analysis results. This information can also help develop threshold criteria for sample exclusion based on DNA yield and quality. Additionally, the methods used for quantification, such as UV-Vis spectrophotometry and fluorescence-based assays, can influence the perceived quality and quantity of DNA. UV-Vis spectrophotometry, while useful for assessing purity, can overestimate DNA concentration due to the presence of contaminants. Fluorescence-based methods, such as Qubit, are more sensitive and specific for double-stranded DNA, providing more accurate quantification (128, 129).

The methods in 48 papers (53.9%) mentioned checking the quantity and/or quality of the analysis material at some point. The most frequently used devices were different versions of the Qubit fluorometers (Invitrogen, Thermo Fisher Scientific) (n = 23) and the NanoDrop spectrophotometers (Thermo Fisher Scientific) (n = 14). Other devices included the Agilent TapeStation 4150 system (n = 5) and the Bioanalyzer 2100 (n = 5) (Agilent Technologies), sometimes alongside Qubit (n = 4). Twelve studies checked the extracted DNA with 0.8% or 1.0% gel electrophoresis alone or in combination with Nanodrop (n = 6) or Qubit (n = 2). Other methods included qPCR (n = 2) and the Promega QuantiFluor Fluorescence Quantification System (n = 1). In the remaining 41 papers (46.1%), quantification or quantification was not mentioned.

While over 50% of the papers mentioned quantification in their methods, quantities were mentioned in only 27 (30.3%) of the reviewed papers which are summarized in **Table 3**. As illustrated in this table, 21 of these 27 papers had described their quantification method, and the other 6 had not. Three studies explicitly stated that quantification was done “to ensure that they meet minimum concentration and mass of DNA”, but they did not specify what the threshold was.

**Table 3:** Summary of quantification results as reported in 26 of the reviewed articles.

First author	Sequencing approach	Collection tool	DNA extraction kit	Quantification method	Any quantification result
Li [2022] (71)	shotgun	membrane	MasterPure	qPCR	100 ng DNA per sample was sonicated
Liang [2021] (49)	shotgun	membrane	MasterPure	qPCR	100 ng DNA per sample was sonicated
Wen [2017] (20)	shotgun	membrane	MasterPure	ns	100 ng DNA was sonicated
Chang [2022] (63)	16s Amplicon [V3-V4]	swab - nylon/flocked	Allprep PowerViral	Quant-iT dsDNA BR assay	All samples produced low DNA yields (<2 ng/uL)
Rocha-de-Lossada [2023] (86)	16s Amplicon [ns]	swab - flocked	QIAamp DNA	Agilent TapeStation	An input DNA amount of 0.5 ng was used for library preparation.
Pal [2022] (72)	16s Amplicon [V3-V4]	microcapillary tube	QIAamp DNA Mini	Qubit	DNA concentrations ranged from 2.5 to 5 ng/μL
Kim [2022] (70)	16s Amplicon [V4]	swab – dry cotton	i-genomic Soil DNA Mini	Qubit	DNA in unused negative control swabs was < 0.05 ng /μL
Wang [2023] (90)	16s Amplicon [V3-V4]	swab - cotton	Custom Protocol	agarose gel	DNA was diluted to 1 ng/mL with sterile water
Yan [2020] (40)	16s Amplicon [V3-V4]	swab - dry cotton	PowerMax Soil	agarose gel	DNA was diluted to 1 ng/μL with sterile water
Borroni [2022] (62)	16s Amplicon [V2-4-8 & V3-6, 7-9]	swab - wet nylon/ flocked	QIAamp DNA	Agilent TapeStation	DNA was diluted to 2 ng/mL, and then 2 mL was used for library preparation
Petrillo [2022] (73)	16s Amplicon [V1-V3]	swab	PowerSoil	Qubit	DNA yield was normalized to 1 ng/μL
Delbeke [2022] (65)	16s Amplicon [V3-V4]	swab - nylon/flocked	RNeasy PowerMicrobiome Kit	Qubit	DNA yield ≤0.5 ng/mL in 7 excluded samples
Zhang [2017] (21)	16s Amplicon [V3]	swab - flocked	Custom Protocol	agarose gel	DNA was diluted to 1 ng/mL with sterile water
Romano [2024] (96)	16s Amplicon [V2-4-8 & V3-6, 7-9]	Swab - moistened flocked	QIAamp DNA	Agilent TapeStation	Input DNA amount of 0.5 ng was used for libraries preparation. Equimolar libraries pooled at final concentration of 10 pM.
Dong [2011] (11)	16s Amplicon [V3-V4]	swab - cotton	PowerSoil DNA Isolation Kit	Agilent Bioanalyzer	obtained a pooled sample containing an average of 241 ng total DNA from each volunteer
Li [2021]	16s Amplicon [V4-V5]	swab - cotton	PowerMax Soil DNA Isolation Kit	Agarose Gel	PCR done with 10 ng template DNA
Zhong [2022] (78)	16s Amplicon [V3-V4]	swab - cotton/flocked	HiPure soil DNA kit	ns	PCR done with 100 ng of template DNA
Huang [2016] (17)	16s Amplicon [V3-V4]	swab - cotton	MicroElute Genomic DNA Kit	ns	PCR done with 25 ng of genomic DNA
Ge [2019] (30)	16s Amplicon [V3-V4]	swab - cotton	MicroElute Genomic DNA Kit	ns	PCR done with 25 ng of genomic DNA extract
Liang [2021]	16s Amplicon [V3-V4]	swab - cotton	kit, but not specified	NanoDrop	PCR done with 3 μLof DNA (20 ng/μL) template
Doan [2016] (16)	16s Amplicon [v3-v4]	swab - nylon/flocked	DNeasy Blood & Tissue Kit	Qubit	PCR done with 5 ng template DNA
Ren [2021] (54)	16s Amplicon [V3-V4]	swab - soaked	DNA extraction kit	agarose gel & NanoDrop	PCR done with 50 ng diluted to 1 ng/mL as template
Zhu [2021] (59)	16s Amplicon [v3-v4]	swab - cotton	MasterPureTM Complete DNA and RNA purification Kit	agarose gel & NanoDrop 2000	PCR done with 50 ng of template DNA
Ji [2022] (69)	16s Amplicon [V3-V4]	swab - nylon/flocked	PowerSoil	Qubit 2 & Agilent Bioanalyzer 2100	PCR done with approximately 10 ng of template DNA
Shivaji [2021] (55)	16s Amplicon [V3-V4]	swab - nylon/flocked - moistened	QIAamp DNA Mini Kit	ns	PCR done with approximately 50 ng template DNA
Li [2019]	16s Amplicon [V3-V4]	swab - cotton	MinkaGene Bacterial DNA Kit	NanoDrop	RCR done with 3 μL of DNA (20 ng/μL) template
Dong [2019] (29)	16s Amplicon [V3-V4]	swab - cotton	PowerMax Soil DNA Isolation Kit	ns	between 11.4 and 80.2 (no measurement unit)

#### 3.4.3 Sequencing approach

Different sequencing methodologies, such as shotgun sequencing and amplicon sequencing, can contribute to variability in results. Shotgun sequencing, while offering species-level resolution and functional insights, is constrained by the high DNA input required and the dominance of host DNA in low-biomass samples, which can inflate costs and reduce usable microbial reads. Amplicon sequencing, which targets specific regions of the 16S rRNA gene, is widely used due to its cost-effectiveness and ability to provide detailed taxonomic information. However, the choice of amplified region can significantly impact microbiome characterization as different hyper-variable regions of the 16S rRNA gene, exhibit varying resolutions and specificities for different bacterial taxa. Consequently, the choice of target region can influence the captured microbial diversity and composition (21, 23, 28). As there is currently no evidence on which 16S rRNA region is the most suitable for sequencing of the ocular surface microbiome, further investigation is required (8).

In 77 (86.5%) of the reviewed studies, amplicon sequencing of the 16S rRNA gene was performed, and the remaining five studies conducted shotgun sequencing (n = 11) or RNA-seq (n = 1). In the former group, the target regions were V1-V2 (n = 3), V1-V3 (n = 4), V3 (n = 2), V3-V4 (n = 44), V4 (n = 11), V4-V5 (n = 4), V5-V6 (n =1), or the full or almost full length of the gene (n =5); the 16S region was not specified in the other 3 studies. Among the 44 studies that targeted the V3-V4 region, some provided the primer name (n = 25) (**Table 4**), primer sequences (n = 29), or both name and sequence (n = 21); primers were not mentioned or specified in the other 11 articles.

**Table 4:** Various primers used for targeting the V3-V4 region of the 16s rRNA gene for amplicon sequencing.

Stated name F	Stated name R	N of articles	Ref #
338F	806R	6	(41, 62, 65, 81, 83, 93)
319F	806R	4	(18, 31, 75, 92)
314F	806R	3	(34, 60, 91)
341F	806R	3	(70, 79, 94)
341F	805R	2	(38, 76)
343F	798R	2	(52, 55)
341F	785R	2	(66, 96)
341F	907R	1	(14)
PSL* forward	PSR* reverse	1	(12)
slightly modified 338F	slightly modified 806R	1	(30)
	Total	25	

*expanded version not found in the paper.

## 4 Discussion

In this review, we examined various methodological aspects of ocular surface microbiome studies, specifically focusing on various aspects of study scope, sample collection and handling, sample preservation, and sample analysis. Our synthesis of this body of literature highlights several key challenges and considerations in the design and reporting of these studies.

### 4.1 Sample size and choice of eye

The absence of sample size calculations was a common finding among the reviewed papers, which can be attributed to various factors. Early studies in the ocular surface microbiome field often lacked access to robust statistical tools or established guidelines for sample size planning. In many cases, sample sizes may have been based on practical considerations such as the availability of participants and resource, rather than formal power analyses. These pragmatic factors, while necessary in some contexts, often resulted in smaller sample sizes without clear justification. Additionally, the exploratory nature of some studies, which focused on generating preliminary data rather than testing formal hypotheses, meant that sample size calculations were not prioritized. This approach was particularly common in earlier work, where small samples were justified by the study’s pilot scope or preliminary descriptive aims. Notably, descriptions of sample size calculations have shown an increasing trend in recent years and improved from 10.5% in 2021 to 22.2% in 2022, 30.8% in 2023, and 50% in 2024.

Given the complexities of microbiome data, particularly for environments with low bacterial biomass, like the ocular surface, the development of reliable and well-validated power analysis tools is an active area of research. Some researchers have suggested a minimum sample size of 30 per group to achieve a statistical power of 0.8 at a significance level of 0.05 (39, 64, 80). However, exceeding these estimates is often necessary due to factors such as individual variability, technical inconsistencies, subgroup analyses, and potential sample loss from low DNA yields. Standardized protocols for reporting and justifying sample size calculations are critical to ensure methodological rigor and reproducibility across future studies.

This review also reveals that while bilateral sampling might seem advantageous for increasing sample size, the potential correlation between the microbiota of the two eyes requires statistical adjustments to avoid skewing results. Additionally, cross-contamination can occur if fellow eyes are sampled with the same swab (92), potentially compromising data integrity and participant safety; adopting separate swabs for each eye can mitigate this risk. As suggested by some of the papers included in this review, although there is significant diversity among individuals, there is a high degree of similarity between the left and right eye microbiome within individuals (11, 16, 20, 22, 46, 63, 88, 130). The similarity between fellow eyes has been observed even in cases of unilateral keratitis compared to normal controls (44). Therefore, while bilateral sampling of eyes is an easy way to double the sample size, it requires adjustments to the sample size to account for the design effect and using statistical methods that can correctly account for the fellow-eye correlation (103). Transparency in reporting sampling strategies—including whether sampling was unilateral or bilateral—and the statistical methods applied is essential for data interpretation and reproducibility. Furthermore, while bilateral sampling presents challenges, it also offers opportunities. Pooling samples from both eyes could potentially increase microbiome yield, provided this approach aligns with the study objectives and mitigates risks such as contamination.

### 4.2 Time frame

The inconsistent and often absent reporting of temporal factors, such as study period and duration as well as sampling time of day, poses a significant challenge to the interpretation and comparison of ocular surface microbiome studies. Variations in environmental conditions or tear film dynamics across seasons and times of day can yield divergent results. For instance, Zhou et al. (14) conducted a 27-month study in the Gambia, revealing significant differences in conjunctival microbiome composition between dry and wet seasons. Such findings highlight the impact of temporal factors and the need for careful documentation.

In this review, nearly 50% of studies failed to report key temporal details, such as sampling times, and the wide range of study durations (2 weeks to 28 months) further complicates comparisons. Short-term studies may capture very different microbial dynamics compared to longer studies, underscoring the importance of standardizing and reporting temporal parameters. Moreover, only four articles (4.5%) specified their sample collection time of the day. This raises important questions about potential biases. Morning samples, collected after a period of eye closure and reduced blinking, might reflect a distinct microbial environment characterized by accumulated secretions, potentially lower oxygen levels, and the absence of daytime mechanical clearance. Conversely, samples taken later in the day would reflect cumulative exposure to the external environment and the ongoing influence of tear flow and blinking. This lack of consistent reporting about the time of day represents another significant gap in our understanding of the ocular surface microbiome.

To address these challenges, we recommend the precise reporting of details of the study time frame—including time of day, time of year, and study duration—as a critical step for transparency and reproducibility. For case-control studies, using time-matched controls, where case and control samples are collected on the same day or within the same week, can further enhance comparability. Additionally, longitudinal study designs should be prioritized to track changes in the ocular surface microbiome over time, offering a more comprehensive understanding of temporal dynamics. Finally, incorporating time as a covariate in statistical analyses can help account for temporal variations and improve the reliability of findings.

### 4.3 Recruitment and enrollment criteria

Our review revealed that convenience sampling was common. While the recruitment process for cases in the case-control studies (n = 66) often involved patients from clinical settings, recruitment strategies for normal controls were often less transparent, complicating the comparability of groups. We also observed significant inconsistencies in the reporting and application of inclusion and exclusion criteria.

Some of the most common exclusion criteria were recent contact lens wear, use of antibiotics or ocular medications, ocular or systemic disease, and history of ocular surgery, and the lack of explicit rationales does not diminish the importance of these criteria. Other potential confounders not used in the reviewed studies include lifestyle factors (e.g., swimming in chlorinated pools, hot tub use) (131), external exposures (e.g., UV radiation, chemical exposure) (132), pet ownership, personal habits (e.g., sleeping position, eye rubbing), dietary habits, cosmetic use (133), and microbiota from other body sites (e.g., skin and gut). However, limited information exists about the specific influence of these factors on the ocular surface microbiome. Therefore, even if not applied as exclusion criteria, it is important to collect information about such potential confounders, study their effects, and if applicable, statistically adjust for their influence to strengthen the study’s internal validity and generalizability. Moving forward, studies can enhance transparency by briefly explaining the biological or methodological significance of their inclusion and exclusion criteria to facilitate interpretation and reproducibility of findings.

Selection of matched controls was stated in only 21 of the 66 case-control studies (31.8%), where age and sex were the most frequent matching criteria. Another important factor that should be considered is the environment. Individuals experiencing similar exposures through shared offices, diet, bedding, or household conditions are likely to have more comparable ocular surface microbiomes. However, our review highlighted inconsistencies in how environmental factors were considered. For instance, while some studies excluded healthcare workers or individuals with recent hospital stays, others recruited participants from these populations. This discrepancy points to a lack of standardized approaches to controlling environmental influences and matching controls.

### 4.4 Sampling environment

This review revealed that negative controls were included in 47.2% of studies, proving valuable in identifying contaminants introduced during sampling. However, 71.2% of studies did not document the environmental conditions of sample collection, signaling a major gap in methodological transparency. Moreover, the variability in reported sampling settings—from sterile operating rooms to participant homes—highlights an absence of universal guidelines. Such inconsistencies may significantly affect microbiome results, necessitating a call for more uniform standards across studies.

While 47.2% of studies included negative controls, a significant portion did not report whether contaminants were detected in these controls. This lack of reporting leaves ambiguity regarding the thoroughness of contamination assessments. Notably, several studies mentioned the use of computational tools for contamination removal, but without clear documentation of their findings from negative controls, it would be challenging to assess the extent to which contamination influenced their results. Furthermore, although tools such as DECONTAM are valuable for *post hoc* contamination filtering, they cannot fully replace the robust inclusion and thorough analysis of negative controls during the experimental phase. Future work should prioritize incorporating negative controls consistently through all experimental phases and reporting results transparently.

### 4.5 Topical anesthesia

Topical anesthetics, such as proparacaine, tetracaine, and oxybuprocaine, are frequently used during ocular surface sampling to minimize discomfort and control reflexive blinking, thereby facilitating consistent sample collection and reducing contamination risks. However, studies have suggested that anesthetics can influence microbial composition, with observed reductions in diversity and selective effects on specific taxa (18, 65, 113, 114). Nonetheless, inconsistent reporting of anesthetic use was noted across the reviewed studies. Furthermore, the use of anesthetics may inadvertently lead to more aggressive sampling techniques and increasing patient risk. This risk extends beyond patient safety, as variations in swabbing pressure, as demonstrated by Dong et al. (11), can significantly alter microbial profiles in favor or a higher abundance of Proteobacteria. To address these concerns, future research should systematically evaluate the effects of anesthetics on ocular microbiome results. Standardized protocols that account for these variables as well as transparent reporting practices are needed.

### 4.6 Collection tool and ocular region

The selection of appropriate collection tools and sampling techniques influences the accuracy and reproducibility of ocular microbiome research. As highlighted by several investigators, variations in swab material (cotton versus flocked nylon) (115) and preparation (dry versus moist) (116) significantly impact results. This is also demonstrated in an experimental study by Wise et al. (134) study, which quantitatively demonstrated the superior DNA recovery of flocked swabs compared to cotton and other materials across various surfaces. Their findings, particularly the poor performance of cotton swabs, which were used in 39 (43.8%) of the reviewed studies, underscore the potential for substantial bias. Moreover, even with flocked swabs, recovery appears to be surface-specific, as evidenced by diminished performance on plastic (134), suggesting that the complex and varied ocular surface may present unique challenges. Future research should include comparative studies of collection methods and the development of evidence-based guidelines to ensure robust and reliable data.

Beyond the choice of collection tool, the specific ocular region sampled appears to influence microbiome analysis. The ocular surface presents a mosaic of distinct microenvironments, and the palpebral conjunctiva, bulbar conjunctiva, fornix, lid margin, and caruncle may harbor unique microbial communities. There are also differences in the ease with which these regions can be sampled, which may introduce inconsistencies; for instance, the fornix, due to its anatomical structure, may be more difficult to swab consistently compared to the relatively flat bulbar conjunctiva. Another consideration is the risk of contamination from the adjacent lid margin and skin, as these areas harbor distinct microbial communities, including skin-associated bacteria which can easily be transferred during swabbing. Additionally, the direction of tear flow, which generally moves from the temporal to the nasal side of the eye, creates a washing effect across the ocular surface, potentially influencing the distribution and abundance of microbes. To fully understand the differences between these regions, researchers must provide detailed descriptions of their sampling techniques, including exact location, depth, and method of collection. Future research should prioritize detailed reporting of the sampled region and its characteristics, ensuring reproducibility and enabling meaningful comparisons across studies.

### 4.7 Sample preservation

Published ocular microbiome studies exhibit significant variability in the way in which samples are preserved, ranging from diverse transport media to inconsistent temperature controls, which can undermine data reliability. Studies in other microbiome research fields, particularly those with high bacterial biomass like stool, suggest that freezing at -80 °C is the best preservation method when immediate DNA extraction is not possible, with some researchers suggesting freezing without buffer as potentially safer for certain sample types (119–122). However, the inherently low bacterial biomass of the ocular surface likely necessitates a more nuanced approach to preservation compared to high-biomass samples. For instance, the sparse ocular microbiota might be more vulnerable to even minor degradation or selective lysis due to transport media. The higher ratio of host to bacterial DNA could also be differentially affected by preservation methods. While stabilization buffers may limit microbial overgrowth at room temperature, they could also disproportionately alter ocular microbial community profiles compared to immediate freezing. There is a need for further comparative studies to identify best practices and tailored preservation techniques for ocular samples. Recommendations include systematic comparisons of preservation methods, the development of standardized guidelines, transparent reporting of all preservation conditions, and the adaptation of methods to the specific needs of each study to enhance the accuracy and comparability of ocular microbiome research.

### 4.8 DNA extraction

In close to 90% of the reviewed articles, DNA extraction was conducted using a commercial kit. However, we identified 40 distinct kits in these 78 articles (**Table 2**), and no single kit emerged as dominant. Notably, some studies applied kits designed for soil samples, which are optimized for high-diversity, high-biomass environments and may not be a suitable choice for the low-biomass ocular surface. This variability, which likely reflects a combination of factors such as individual preferences, kit availability at the time, ease of use, cost considerations, and sample type (e.g., swab, tissue, fluid), could introduce biases that affect DNA yield, quality, and microbiome composition, complicating cross-study comparisons. However, the limited availability of quantity and quality check results (see Checking the Extracted DNA) in the reviewed studies prevents any direct assessment of how extraction protocols may influence DNA yield or quality.

### 4.9 Checking the extracted DNA

One of the most significant gaps identified in this review was the inconsistent reporting of DNA quantities. While most studies mentioned checking their DNA, the actual yields were rarely provided. Among the few studies that reported quantification results, DNA yields varied considerably. Chang et al. (63) reported that all their samples yielded less than 2 ng/μL, while Pal et al. (72) documented DNA concentrations ranging from 2.5 to 5 ng/μL. In another study, Delbeke et al. (65), who incorporated an additional lysis step in their DNA extraction protocol, excluded about 30% of their samples due to DNA yields of ≤0.5 ng/mL (0.0005 ng/μL) being too low for sequencing. It is likely that other studies encountered similar issues with low-yield samples, but these were not explicitly reported, highlighting another significant gap in the literature.

A common practice, however, was to report standardized target input DNA amounts used for downstream PCR or library preparation, such as 0.5 ng, 10 ng, 25 ng, 50 ng, or even 100 ng, rather than the initial DNA yield. This approach, which may reflect adherence to pre-established protocols focused on achieving consistent input for downstream steps, effectively obscures the true amount of DNA extracted from the ocular surface. While quantification was presumably performed on the extracted DNA to determine the volume needed to reach these target input amounts, the crucial information regarding the initial DNA recovery from the samples was not consistently reported.

Some research demonstrated successful downstream analyses using input DNA amounts as low as 0.5 ng (86) or even pico-molar concentrations (96), adding to the complexity of interpreting results. Additionally, certain studies reported diluting their samples to 1 ng/mL (54, 57) or 2 ng/mL (62) concentrations, making it challenging to determine the original extracted amounts. This variability, combined with the frequent use of standardized input amounts instead of reporting initial yields, highlights the need for standardized reporting practices and protocols in ocular microbiome research.

### 4.10 Sequencing approach

Another gap identified in this review was the lack of consensus on the optimal 16S rRNA region for sequencing the ocular surface microbiome. While the V3-V4 region was the most frequently targeted (44 out of 89 studies), no definitive evidence supports its superiority over other regions, such as V1-V3 or V4-V5, in capturing microbial diversity. Nonetheless, the predominance of V3-V4 region may be an opportunity for meta-analyses to examine how the variability observed in methodologies may influence results in ocular microbiome research.

## 5 Summary of methodological and reporting considerations

To synthesize the key methodological challenges identified in this review, **Table 5** summarizes common sources of variability and corresponding considerations for improving rigor and reproducibility in ocular surface microbiome research. In addition to methodological diversity, we found incomplete reporting to be a major barrier to transparency and comparability. A few studies provided relatively more comprehensive documentation across critical parameters. For example,

**Table 5:** Summary of methodological considerations.

Step	Common variability	Key considerations
Sample Size	Often missing or pilot-based	Report calculations and rationale; account for low biomass
Choice of Eye	Unilateral vs bilateral; pooling vs independent	Adjust for correlation; report strategy clearly
Temporal Factors	Duration, season, time of day	Standardize reporting; use time-matched controls
Recruitment Criteria	Convenience sampling; unclear exclusions	Define and justify inclusion/exclusion; note confounders
Sampling Environment	Sterile vs non-sterile; negative controls absent	Document conditions; include and analyze negative controls
Topical Anesthesia	Proparacaine, tetracaine, oxybuprocaine	Report use and type; assess potential microbial impact
Collection Tools	Swab vs paper; cotton vs nylon; flocked vs spun; moist vs dry	Specify material, tip type, preparation, evaluate tool performance
Sample Preservation	Media and temperature vary widely	Report all conditions; standardize where possible
DNA Extraction	40+ kits used; modifications applied	Report kit, modifications, and DNA yield
Sequencing Approach	V3–V4 most common; other regions used	Report region and primers; justify choice

– Jiang et al. (25) reported start–end dates, recruitment source of both patients and healthy controls, sampling environment (sterile operating room), bilateral sampling (independent), no anesthesia, and included negative controls (unused swabs exposed to operating room air).
– Andersson et al. (43) reported start–end dates, explained why sample size was not calculated (pilot; lack of previous studies), used bilateral pooled sampling, no anesthesia, and included negative controls (unused swabs).
– Li et al. (71) documented timeframe, justified lack of sample size calculation (“no formal null hypothesis”), used unilateral sampling (right eye), anesthesia (Alcaine), and negative controls (unused membranes).
– Suwajanakorn et al. (75) reported start–end dates, calculated sample size (formula comparing two independent means), used unilateral sampling (right eye), anesthesia (tetracaine), and negative controls (unused swabs).
– Romano et al. (96) (2024) calculated sample size (based on 20% microbial load reduction, 80% power, 5% alpha), used independent bilateral sampling (contralateral control), no anesthesia, the Copan ESwab collection device (a tube with 1 mL of Amies medium and a flocked swab), and PCR-based negative controls.

Although these studies varied significantly in their methodological choices, they represent stronger reporting practices compared to most reviewed articles. Spotlighting such examples emphasizes that transparent documentation – even when methodological diversity persists – is essential for reproducibility and cross-study comparisons.

## 6 Conclusion

This review underscores the significant methodological variability and reporting inconsistencies prevalent in ocular surface microbiome studies. From temporal considerations and sample size calculations to DNA extraction and sequencing approaches, numerous factors may influence study outcomes, yet are not adequately reported in the literature, and are potentially ignored in study design. The lack of standardized protocols and transparent reporting practices impedes the comparability and reproducibility of research findings. Moving forward, the field must prioritize the development and adoption of evidence-based guidelines, emphasizing meticulous documentation of all experimental parameters. This includes detailed reporting of temporal factors, rigorous sample size justifications, standardized sampling and preservation methods, and comprehensive documentation of DNA extraction and sequencing procedures. By addressing these methodological gaps, future research can enhance the reliability and generalizability of ocular microbiome studies, supporting a deeper understanding of the ocular surface ecosystem and its implications for ocular health and disease.

## 7 Limitations

This review focused exclusively on summarizing sample collection methods and did not analyze study outcomes or results. Consequently, the synthesis does not capture potential associations between collection techniques and microbiome profiles. Additionally, the review may not include all relevant studies due to language restrictions and database coverage, which could introduce selection bias. Variability in reporting details across the reviewed studies also limited our ability to fully compare protocols.
